# Stress, Mindsets, and Success in Navy SEALs Special Warfare Training

**DOI:** 10.3389/fpsyg.2019.02962

**Published:** 2020-01-15

**Authors:** Eric N. Smith, Michael D. Young, Alia J. Crum

**Affiliations:** ^1^Department of Psychology, Stanford University, Stanford, CA, United States; ^2^Warfighter Performance, Naval Health Research Center, San Diego, CA, United States

**Keywords:** mindsets, stress-is-enhancing, non-limited willpower, failure-is-enhancing, military, persistence, multiverse analysis

## Abstract

Mindsets can impact an individual’s performance in stressful experiences such as public speaking or receiving negative feedback. Yet we know little about the boundary conditions of where these mindsets predict success, and where they may become irrelevant or even maladaptive. The current research asks whether mindsets are beneficial in environments of extreme physical and mental stress using participants undergoing the notoriously challenging Navy SEALs training. We hypothesized that participants with stress-is-enhancing mindsets – who believe stress enhances their health, performance and wellbeing – will outperform those with stress-is-debilitating mindsets. In addition, we explore whether other mindsets about willpower and failure predict success in a similar manner. Following 174 Navy SEALs candidates, we find that, even in this extreme setting, stress-is-enhancing mindsets predict greater persistence through training, faster obstacle course times, and fewer negative evaluations from peers and instructors. We also find evidence that failure-is-enhancing mindsets may be detrimental to candidates’ success, and non-limited willpower mindsets prompt negative evaluations from others. Multiverse analyses were conducted to test for the robustness of these effects across researcher analytical decisions, which produced consistent results. We discuss how findings in this unique environment can provide insight into the importance of mindsets in other organizations and propose future avenues of research to further understand the causal role of mindsets in diverse workplace contexts.

## Introduction

Stress is pervasive in modern life, affecting everything from work performance and cognitive ability to mental and physical health (Motowidlo et al., 1986; McEwen and Sapolsky, 1995; Schneiderman et al., 2005). Though a majority of the existing literature has focused on documenting the negative aspects of stress, researchers from a variety of disciplines have shown that stress can lead to adaptive physiological and psychological functioning (Epel et al., 1998; Tedeschi and Calhoun, 2004; Park and Helgeson, 2006; Duncko et al., 2007). Increased stress has also been linked to improved performance during physically and mentally demanding tasks (LePine et al., 2005; Jones et al., 2009). Organizations and individuals may find that stress can increase efforts and improve performance in some instances, but hinder performance in others (see Podsakoff et al., 2007 for review).

Yet it is not simply the true nature of stress that can enhance or undermine functioning, but also the mindsets people hold about stress (Blascovich and Mendes, 2010; Jamieson et al., 2010; Crum et al., 2013). Mindsets are the core assumptions about the nature of many different things and processes in the world, which orient people to a particular set of expectations, attributions, and goals (adapted from Molden and Dweck, 2006; see also Zion et al., 2019). Mindsets have shown to impact a variety of real-world outcomes including academic performance, well-being, and health (Crum et al., 2013; Romero et al., 2014; Yeager et al., 2014; Paunesku et al., 2015). In the domain of stress, some people believe stress enhances health, performance and wellbeing (“stress-is-enhancing”) while others believe that stress has generally debilitating consequences (“stress-is-debilitating”). People with stress-is-enhancing mindsets or those given information about the positive aspects of stress have more optimal physiological responses to stressors and report fewer negative health symptoms and greater positive emotions (Crum et al., 2013, 2017). Moreover, employees at a large finance institution, which at the time was undergoing downsizing in the wake of the 2008 recession, reported increased work performance after learning about the positive aspects of stress (Crum et al., 2013), suggesting stress-is-enhancing mindsets allow employees to better sustain investment during challenge and uncertainty.

Although this past work on stress mindsets is promising, some key questions have been left unanswered. First, much of the prior work has relied on self-report outcome measures or performance during one task. As stress-is-enhancing mindsets are related to improved emotional outcomes (Crum et al., 2017), it is unclear whether self-report improvements reflect true change, or biased reporting due to increased positive affect (see Podsakoff et al., 2003). In addition, self-report is prone to social desirability effects and methodological artifacts (Podsakoff and Organ, 1986). For research examining objective performance, studies have generally been limited to one task or exam (Brooks, 2014; Akinola et al., 2016; Crum et al., 2017). By collecting longitudinal, objective measures of performance, we can start to understand whether stress mindsets are important to holistic functioning and achievement.

Second, we do not yet know the boundary conditions of these mindsets. In environments with prolonged and high-intensity demands, are stress mindsets still beneficial? Some research has suggested that these mindsets may be even more impactful during extreme stress (Akinola et al., 2016; Park et al., 2017). Other research has suggested the opposite – that mindsets cannot override physiological limits during extreme challenges (Vohs et al., 2012). We do not yet know where mindsets have the most impact, and where they may become irrelevant or maladaptive to success.

Furthermore, stress mindsets may not be the only mindsets at play in extremely challenging settings – mindsets surrounding failure and willpower may also play a role. Failure-is-enhancing mindsets tap into the belief that failure can increase learning, growth, and performance, in a similar manner as stress-is-enhancing mindsets. Although little work has examined failure mindsets in particular (see Haimovitz and Dweck, 2016 for exception), related beliefs have been a core component of implicit theories of growth, in which failures indicate an opportunity to learn from mistakes (Dweck and Leggett, 1988; Mueller and Dweck, 1998). The majority of research has found these failure-relevant growth mindsets promote long-term success (e.g., Blackwell et al., 2007), yet there is evidence for the opposite relationship. When participants failed on a task where growth and improvement were seen as possible, they put less effort into future tasks compared to a performance-oriented task where ability was purportedly static (El-Alayli and Baumgardner, 2003; see also Harackiewicz et al., 2000; El-Alayli, 2006).

A non-limited willpower mindset – the belief that willpower and energy are maintained or enhanced with effort, rather than limited or drained – may also be highly relevant to extremely stressful settings. Research has found that people vary in the extent they believe willpower is non-limited and that those with non-limited theories of willpower make fewer mistakes during demanding tasks (Job et al., 2010), are less sensitive to physiological constraints that might hold them back (Job et al., 2013), and use their time and energy more adaptively (Job et al., 2015).

All three of these mindsets appear to be beneficial in stressful or challenging settings, but the boundary conditions of these mindsets are unclear. To determine the potential limits of these mindsets, we identified a population that undergoes extreme levels of stress, and where objective performance data and persistence can be tracked over the course of weeks: candidates in Navy SEAL elite military training. As theorized in previous literature (e.g., Crum et al., 2013), we hypothesize that stress-is-enhancing mindsets collected prior to training will predict persistence, objective metrics of performance, and subjective social ratings from others in this setting. We also examine whether failure and willpower mindsets uniquely predict the same outcomes or differentially impact success.

## Materials and Methods

### Population

United States Navy SEALs (named for the settings they operate in: Sea, Air, and Land) are an elite military force responsible for special operations, working in chaotic and unknown environments with little margin for error. Some of the most notable missions in recent history including the rescuing of a cargo ship from Somali pirates in 2009 and the killing of Osama bin Laden in 2011.

Given the high stakes of these missions, it is no wonder that training to become a Navy SEAL is known to be one of the most challenging training regimens in the world. A centerpiece of this training involves the completion of Basic Underwater Demolition/SEAL (BUD/S) First Phase, in which candidates undergo 7 weeks of intense physical and mental training. The most grueling part of this training takes place in week 4, known simply as “Hell week,” in which candidates complete tasks and drills throughout nearly non-stop five and a half days of training, while undergoing extreme sleep deprivation (receiving approximately 45 min of sleep per night). In recent years, 7–20% of candidates who start BUD/S training successfully complete it. Candidates drop due to a variety of reasons, including medical injuries, not reaching performance standards, or “dropping on request,” by opting to leave BUD/S during training.

Candidates’ stress mindsets may be particularly important in this extremely stressful setting. Instructors convey stress-relevant messages about welcoming stress in training (colloquially referred to as “embrace the suck”) and simultaneously attempt to increase candidates’ stress throughout training to mimic combat settings. Candidates who see stress as beneficial may show greater persistence and performance throughout training. Those who feel stress is taking a toll on their physical and mental wellbeing, or reducing their potential for success, may feel unable to cope with the continuously increasing demands.

Although this population exemplifies the extreme of stress-inducing work, in any organization the performance of individuals depends in part on their mindsets – what their stress, efforts, and failures indicate about their ability to succeed in that setting. As individual and group-level expectations for performance are a robust predictor of actual performance (e.g., Stajkovic and Luthans, 1998; Miao et al., 2017), understanding the role of mindsets can shed light on how to support employees during acute or chronic stressors that impact their work life.

### Participants

**FIGURE 1:**
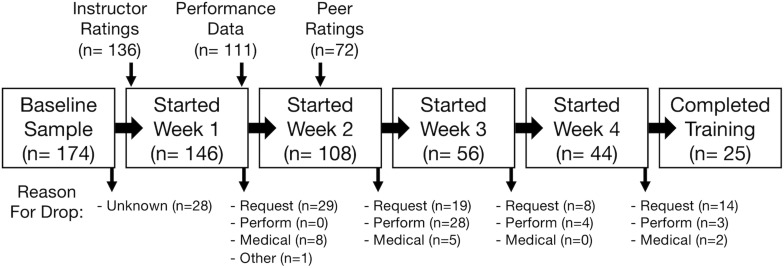
Number of participants through training and reported in outcome variables. Reasons for dropping from the cohort are also provided. All candidates who completed Week 4 completed all 7 weeks of training. Request = Dropped on request; Perform = Dropped due to performance; Medical = Dropped due to a medical illness or injury. Reasons for dropping prior to week 1 were not recorded.

A cohort of Navy candidates from across the United States, previously screened and selected for BUD/S, participated in this research. Each cohort of candidates starts orientation approximately every 2 months. Candidates from one cohort may be “rolled” to the next cohort if instructors believe the candidate has potential but is not able to complete training with their current class. We only include candidates going through the orientation phase and exclude other candidates rolled into this class during phase one. Participants included both enlisted men and officers undergoing the same training. Participants left the training throughout data collection, and the number of participants in analyses vary across measures (see Figure 1). We initially intended to recruit 300 participants across two cohorts of BUD/S candidates. However, due to logistical constraints, we were only able to recruit one cohort of 174 BUD/S candidates, allowing us to detect small effects of β = 0.20 with approximately 75% power. Of the 174 candidates who consented to participate in the study during the orientation phase of BUD/S, 146 started the official first day of training, 45 started week 4 of training (“Hell Week”), and 25 successfully graduated phase 1 after completing all 7 weeks, which is consistent with completion rates of recent cohorts (14% in the current class compared to ∼7–20% in recent years). All participants were Male (training recently opened up to women after data were collected), 85% reported being White/Caucasian, and 45% had completed a 4-year college degree.

### Procedure

During orientation prior to BUD/S training, the research team visited the naval base to introduce the research and survey to potential participants. Candidates were told verbally about the study prior to participation and were given one hour between training activities to consent to participate in the study and complete a baseline survey if they chose to do so. We clarified that participation was optional, participating would not affect their training, and that their military supervisors would not have access to their responses. We also asked their instructors to leave the room to minimize any possibility of coercion or candidates believing this was a required aspect of training. Of the 176 surveys handed out, 174 were returned with a consent form completed. Upon the cohort completing phase one, instructors securely transferred participant performance and persistence data to the research team.

Participants were also given follow-up surveys by the research team prior to the fourth week of training, and upon completion of training or removal from the class to assess how candidates’ mindsets changed across training. For the purposes of the current study, we focus on the predictive power of measures collected at baseline. All measures collected, additional information about follow-up surveys, and analysis details can be found in the Supplementary Material.

### Baseline Survey Measures

#### Demographic Information

Participants completed demographic information on their personal education level (0 = Some college or below; 1 = College degree or higher), parental education level (0 = Some college or below; 1 = College degree or higher), Race (0 = Non-White; 1 = White), and Body Mass Index (BMI; *Weight-in-pounds/Height-in-inches^2^ × 703*). Missing data were imputed to the mean value for each demographic measure to include all participants in analyses.

#### Stress Mindset Measure

A person’s stress mindset is the extent to which a person holds the belief that stress has debilitating outcomes (a “stress-is-debilitating mindset”) or believes that stress has performance-enhancing outcomes for health, learning, and growth (a “stress-is-enhancing mindset”). We used the eight-item Stress Mindset Measure, which has adequate reliability and psychometric properties (Crum et al., 2013). Participants were asked to indicate their agreement to items such as “The effects of stress are positive and should be utilized” (1 = “Strongly Disagree”; 6 = “Strongly Agree”). Four negatively worded items were reverse-coded and all items were averaged to form a composite scale (α = 0.70).

#### Failure-is-Enhancing Mindset

This six-item failure mindset scale was directly adapted from the Stress Mindset Measures above, replacing “Stress” with “Failure” (Haimovitz and Dweck, 2016). Participants indicated their agreement to items such as “Experiencing failure improves performance and productivity” (1 = “Strongly Disagree”; 6 = “Strongly Agree”). Three negatively worded items were reverse-coded and all items were averaged to form a composite scale (α = 0.82).

#### Willpower Mindset

This six-item scale taps into the belief that people can access extra energy after strenuous activity and that willpower is non-limited. This scale has shown adequate reliability and psychometric properties (Job et al., 2010, 2015). Participants were asked to indicate their agreement to items such as, “After a strenuous mental activity, you feel energized for further challenging activities” (1 = “Strongly Disagree”; 6 = “Strongly Agree”). Three negatively worded items were reverse-coded and all items were averaged to form a composite scale (α = 0.77).

#### Additional Predictors of Persistence

Based on discussions with BUD/S instructors, we assessed three additional items that we expected might to be related to candidate success in training. This included whether a candidate was committed to BUD/S (“My goal of becoming a Navy SEAL is more important than any other goal in my life.”; 1 = “Not at all true,” 5 = “Completely true”), the candidate’s optimism for completing training (“How likely do you think it is that you’ll finish BUD/S successfully?”; 1 = “Not at all likely,” 5 = “Extremely likely”), and whether he had a mentor who prepared him for training (“I have a mentor within the Navy that supports my personal development.”; 1 = “Not at all true,” 5 = “Completely true”).

#### Social Desirability

Based on instructors’ and researchers’ observations at the base, we were concerned that some participants responded in socially desirable ways. That is, it appears that many BUD/S candidates believe that everything they do is being tracked and judged by their instructors. We attempted to reduce these concerns by (1) visiting the base in-person to hand-deliver and collect the surveys, and (2) asking instructors to leave the room so that they could not see candidates’ responses. However, upon initial review of the data we found evidence that some participants were answering in socially desirable ways, such as reporting only extreme values on particular scales. For this reason, we created a social desirability metric to ensure our effects were not simply due to socially desired responses on our measures.

To create this social desirability metric, we utilized a subset of 7 items from the 15-item Mindful Attention Awareness Scale (Brown and Ryan, 2003) which we had included in the surveys as an exploratory measure. However, these items were ideal to use as a measure of social desirability, as the “correct” responses of someone presenting themselves in the best light were quite obvious and extreme in a typical population. These items asked participants to respond to the frequency in which they do things such as, “I find myself doing things without paying attention.” Or “I forget a person’s name almost as soon as I’ve been told it for the first time.” Similar items have been used in past research to assess social desirability (e.g., “No matter who I’m talking to, I’m always a good listener.”; Crowne and Marlowe, 1960). We thus calculated a desirability cutoff score based on participants who reported “Almost Never” to at least six of these seven items, and include this dichotomous coding in each model reported below (0 = “Low Social Desirability”; 1 = “High Social Desirability”).

#### Exploratory Measures

Given the unique opportunity of having access to this population of participants, we included exploratory measures of mindfulness and willpower-relevant items, BUD/S-specific beliefs, importance of personal strengths in training, and perceptions of the BUD/S training environment. Open-response items were also collected. All quantitative measures collected can be found in the Supplementary Material.

### Primary Outcomes-Persistence, Success, and Performance

#### Persistence

We were able to collect three main metrics of participants’ success throughout training. First, we assess persistence in training as the amount of time participants successfully stay in BUD/S training before deciding to drop out or left for medical, performance, or other reasons. There was sometimes a delay of up to 3 days between the time of ending training and officially being recorded as removed from the class. For this reason, we use the week in which candidates drop out of the program, rather than the particular day recorded. Participants who dropped out between orientation and the start of orientation were coded as 0, drops from the class in weeks 1–4 were recorded as that week, and all candidates who completed the training were coded as a five. Although the training lasted 7 weeks, no candidates dropped during weeks 5–7, and thus were coded as a five to maintain a more interpretable distribution of outcomes.

#### Success

Second, we assess the binary outcome of whether each candidate successfully completes phase 1 (1 = Graduate; 0 = Non-Graduate). Due to the nature of the training, 15 participants dropped out of the program or “rolled” to the next class due to medical reasons. We exclude these participants in analyses of completion proportions and the weeks persisted measure described above, as medical issues do not necessary reflect the physical or mental toughness of candidates (e.g., medical drops may be due to accident or misfortune).

#### Performance

Third, we collected objective measures of performance in BUD/S. We received completion times for an obstacle course, a four-mile run, and a two-mile swim conducted each week of training. In particular, we look at obstacle course performance during week 1, controlling for other physical performance of run and swim times. The number of participants with performance data was greatly reduced after week 1, so performance measures in future weeks were not analyzed. We hypothesized that mindsets would be more predictive on the obstacle course as it requires both mental and physical stamina in a novel task and relies less solely on previous training and baseline physical fitness compared to run and swim times.

### Secondary Outcomes

#### Peer and Instructor Evaluations

Peer and instructor evaluations were analyzed to determine whether mindsets not only predicted individual success, but also how that individual was viewed by others. Instructors made notes throughout training on candidates, marked whether their comment was positive or negative (+1 or -1), and selected a short description chosen from a pre-defined list on what they observed (e.g., “+1: Cooperative and hardworking”; “−1: Demonstrated less than 100 percent effort”), with an option of providing more detailed notes. As fewer than 2% of instructor comments were positively valenced, candidates tended to accrue negative comments over the course of training. To maximize participants included in the analyses, we remove positive comments and divide instructor negative evaluations by number of weeks in training. Peer ratings were collected at Week 2, with a similar positive, neutral, or negative valence given for each of the 72 candidates remaining, and open-responses to clarify their responses. Seventy candidates filled out these ratings, and the number of positive and negative comments were summed and analyzed separately.

## Results

### Analytic Approach

For each outcome we report a primary linear model based on our theory and best methodological practices. Each model includes all three mindset measures entered simultaneously, such that reported findings signify incremental variance beyond that explained by other mindsets. The primary models are both theory-based, in which our theory influenced the measures collected and exclusion criteria as discussed above, and empirically driven, as covariates in the models were determined by cross-validated LASSO regression analysis with a regularized tuning parameter (Tibshirani, 1996). This LASSO method performs a variable selection to reduce the number of included covariates and minimizes issues using suboptimal variable selection methods such as stepwise regression (McNeish, 2015). We test whether the collected demographic measures, additional predictors of persistence, and the social desirability metric were informative predictors of any of the three primary outcomes. We find that items assessing participants’ education level, parental education level, BMI, optimism for success, and the social desirability metric were informative in at least one of the LASSO models and thus retained as covariates. The primary linear models for each outcome include these covariates. A logistic regression model was conducted for the binary completion outcome with the same predictors.

We adopted this analysis strategy because we believed it to be the strongest test of our hypotheses, but acknowledge that other researchers, if given the opportunity to analyze this dataset, may have adopted one of many different analysis strategies. For example, researchers may have chosen different exclusion criteria, included different covariates, or dealt with missing values in different ways. To ensure our analyses are robust to these possible analytic choices, we also perform *multiverse analyses* (see Steegen et al., 2016) for each outcome. That is, we conducted 104,976 potential analyses (including the primary model) that a researcher could have run in the “multiverse” of analytic models testing for effects on the same outcomes and present the aggregate results of these analyses below (see Table 1 and Supplementary Material for full details). If these aggregate statistics are consistent with our primary model, the multiverse analyses would provide evidence that our findings are robust to the specific analytic decisions or covariates included in our primary models.

**TABLE 1:** Researcher decisions included in multiverse analysis.

**Decision points**	**Multiverse analysis decision options**	**# of models**
**Include demographics?**		
Education	**Impute missing values**/remove missing values (*N* = 8)/do not include in model	3
Mother education	**Impute missing values**/remove missing values (*N* = 9)/do not include in model	3
Body mass index	**Impute missing values**/remove missing values (*N* = 10)/do not include in model	3
Race	Impute missing values/remove missing values (*N* = 9)/**do not include in model**	3
**Include survey covariates?**		
Social desirability^1^	**Include cutoff criteria**/include continuous variable/do not include in model	3
Optimism for success	Impute missing values/**remove missing values (*N* = 2)**/do not include in model	3
Navy mentor^1^	Include in model/**do not include in model**	2
BUD/S commitment	Impute missing values/remove missing values (*N* = 1)/**do not include in model**	3
**Include initial physical performance?**		
Week 1 run & swim times	Impute missing values/remove missing values (*N* = 63)^2^/**do not include in model**	3
**Exclusion criteria?**		
Outliers (>3 SD)	**Include**/exclude (*N* = 3)	2
Medical drops/rolls	Include/**exclude (*N* = 15)**	2
Rolls to next class	**Include**/exclude (*N* = 12)	2
Total models run	(3)^8^ × (2)^4^	104,976

*Primary model choices are bolded as described in the main manuscript and decision points are discussed in further detail in the Supplementary Material. ^1^There were no missing values for these scale such that removing missing values would be equivalent to imputing missing values. ^2^Performance covariates were used only in the primary model predicting week one obstacle course times, as described in text.*

### Baseline Information

#### Baseline Social Desirability

As expected, a portion of participants appeared to show socially desirable reporting. Twenty-three (13%) of the participants reported they almost never engaged in non-mindful behaviors for at least six of the seven items. On the other hand, 121 participants (70%) reported three or fewer items to which they reported never engaging in. We did not necessarily expect Social Desirability to relate to outcomes, but rather to other beliefs for which these participants might put socially desirable responses. We found that social desirability does relate to responses on measures of Stress Mindsets and Willpower Mindsets (*r*s > 0.25; *p*s < 0.001). Interestingly, there was no significant relationship between social desirability and Failure Mindsets (*r* = 0.03, *p* = 0.693), suggesting that these candidates did not know what the socially desirable answers to the failure mindset measure would be from the perspective of instructors.

#### Baseline Survey Correlations

We find that mindsets on stress, failure, and willpower were significantly correlated with each other (*r*s > 0.20, *p*s < 0.007; see Table 2). It is notable that participants reported greater stress-is-enhancing mindsets than populations studied in the past (e.g., Crum et al., 2013; Park et al., 2017). This may be due to self-selection into this extremely stressful environment, social desirability effects, or a combination of these factors.

**TABLE 2:** Means, standard deviations, and correlation coefficients across demographic and baseline measures.

		**Correlation coefficient**		
	**Mean (SD)/ percentage**	**Stress mindset**	**Failure mindset**	**Willpower mindset**
Stress-is-enhancing mindset	4.54 (0.66)	–		
Failure-is-enhancing mindset	4.81 (0.78)	0.33^∗∗∗^	–	
Non-limited willpower mindset	4.50 (0.69)	0.44^∗∗∗^	0.21^∗∗^	–
Race (Non-white)	15%	0.08	0.04	0.05
Education (college degree)	45%	–0.02	–0.04	0.01
Mother education (college degree)	59%	–0.04	–0.08	0.02
Social desirability (cutoff)	13%	0.25^∗∗∗^	0.03	0.30^∗∗∗^
Body mass index	25.0 (1.74)	–0.01	0.02	0.11
Perceived likelihood of success	4.55 (0.75)	0.27^∗∗∗^	0.09	0.19^∗^

*Demographic variables are reported as percentage non-white, college educated, having a college educated mother or guardian, and indicating socially desirable reporting. Pearson correlation coefficients are calculated for all continuous variables and point-biserial correlations are calculated for binomial measures. All questionnaire items were on a six-point scale (1 = “Strongly Disagree”; 6 = “Strongly Agree”). ^∗^*p* < 0.05, ^∗∗^*p* < 0.01, ^∗∗∗^*p* < 0.001.*

### Do Stress-Is-Enhancing Mindsets Predict Success?

#### Persistence, Success, and Performance

**FIGURE 2:**
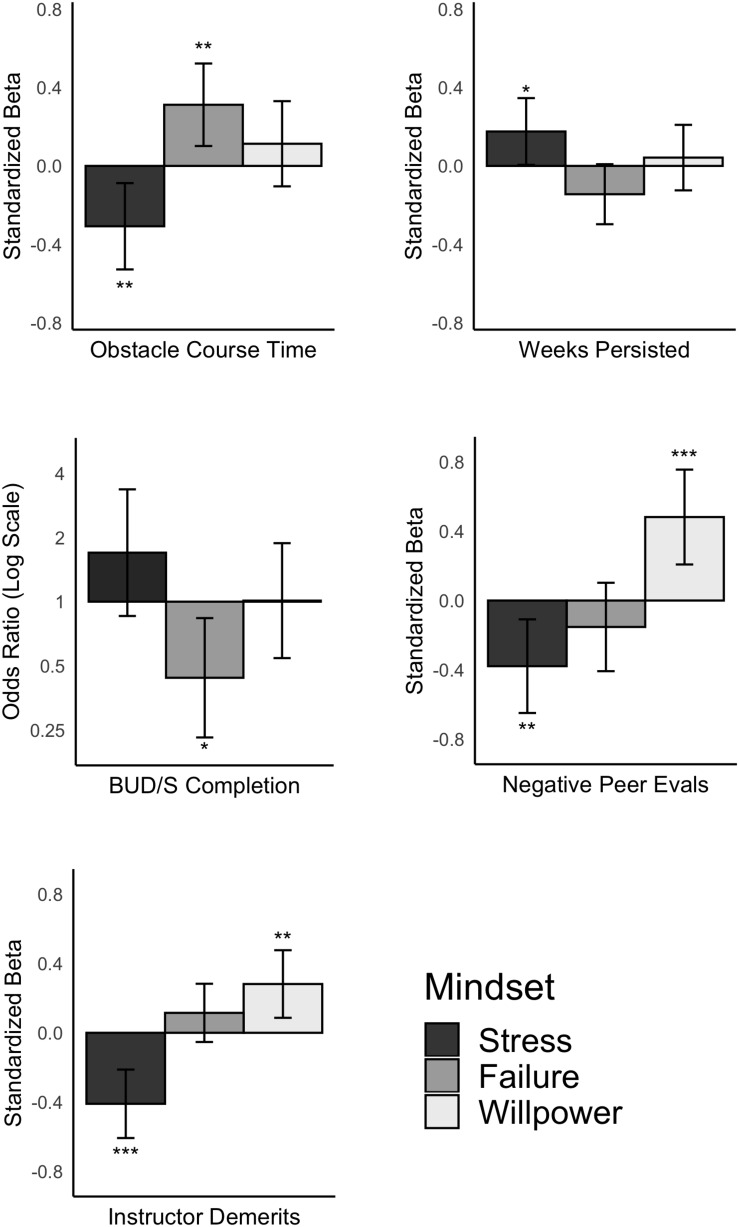
Bar graph comparing effect sizes from the linear models reported in text for each of the collected mindsets on primary and secondary outcomes. Values above zero represent predictors that correspond to higher values of that outcome. Error bars represent 95% confidence intervals. ^∗^*p* < 0.05, ^∗∗^*p* < 0.01, ^∗∗∗^*p* < 0.001.

Using our primary model as described above, we find that BUD/S candidates with a greater stress-is-enhancing mindset (+1 SD) had a better chance of persisting in training compared to those with average stress mindset (12% longer; β = 0.18, *t*(148) = 2.04, *p* = 0.043, 95% CI [0.01, 0.35]) and had a directionally, but not significantly higher chance of completing phase 1 successfully (54% more candidates completed; *OR*(156) = 1.70, *p* = 0.129, 95% CI [0.86, 3.37]; See Figure 2). We also find that candidates with a stress-is-enhancing mindset had faster times on the Obstacle Course by 27 seconds (4.2% faster; β = −0.31, *t*(99) = −2.78, *p* = 0.007, 95% CI [−0.53, −0.09]). The multiverse analyses of 104,976 possible models for each outcome suggested these findings were robust to analytic decisions: averaged across all models, stress mindset was found to significantly predict weeks persisted (β_median_ = 0.19, *p*_median_ = 0.033), and obstacle course performance (β_median_ = −0.29, *p*_median_ = 0.011). The directional non-significant effect on successful completion was also robust across models (*OR*_median_ = 1.63, *p*_median_ = 0.166).

#### Instructor and Peer Evaluations

We find evidence that those with a stress-is-enhancing mindset were seen more positively by both their instructors and peers. Participants with greater enhancing stress mindsets (+1SD) had 30% fewer negative comments per week from instructors compared to the average candidate (β = −0.41, *t*(130) = −4.10, *p* < 0.001, 95% CI [−0.61, −0.21]). We also find that although stress mindset did not predict a greater number of positive evaluations given by other candidates, (15% more, β = 0.14, *t*(62) = 0.92, *p* = 0.361, 95% CI [−0.16, 0.43]) candidates with greater stress-is-enhancing mindsets received significantly fewer negative evaluations by peers (60% fewer, β = −0.38, *t*(62) = −2.80, *p* = 0.007, 95% CI [−0.65, −0.11]). The multiverse analyses suggest these results are analytically robust, finding stress mindsets predicted fewer negative evaluations from instructors (β_median_ = −0.40, *p*_median_ < 0.001) and peers (β_median_ = −0.33, *p*_median_ = 0.008).

### Do Other Mindsets Predict Success?

We also examined the role of failure-is-enhancing mindsets and non-limited willpower mindsets for the same measures reported above. For brevity, we only report significant or marginally significant findings for each mindset (*p*s < 0.10; see Table 3 for results from all primary models).

**TABLE 3:** Standardized effect sizes for outcomes.

	**Primary outcomes**			**Secondary outcomes**		
**Measure**	**Successful completion (logistic)^1^**	**Weeks persisted**	**Obstacle course time**	**Instructor evaluation (negative)**	**Peer evaluation (negative)**	**Peer evaluation (positive)**
*Demographic covariates*						
Education	7.096^∗∗∗^	0.349^∗^	0.235	−0.380^∗^	–0.213	0.393
Mother education	2.185	0.335^∗^	–0.132	–0.189	–0.213	–0.265
Body mass index	1.128	0.151^∗^	–0.089	0.015	–0.127	0.145
*Survey covariates*						
Social desirability	0.000^2^	–0.626^∗∗^	–0.110	0.517	0.399	–0.505
Optimism for success	1.824	0.162^∗^	–0.051	0.035	–0.028	0.156
*Performance covariates*						
4-Mile Run^3^	–	–	0.452^∗∗∗^	–	–	–
2-Mile Swim^3^	–	–	0.102	–	–	–
*Mindsets*						
Stress-is-enhancing	1.699	0.176^∗^	–0.308^∗∗^	–0.410^∗∗∗^	–0.379^∗∗^	0.137
Failure-is-enhancing	0.439^∗^	–0.145	0.312^∗∗^	0.115	–0.153	–0.161
Non-limited willpower	1.011	0.042	0.113	0.282^∗∗^	0.482^∗∗∗^	–0.042

N_obs_	157	157	110	130	71	71
R^2^	–	0.218	0.304	0.196	0.304	0.158

*Education measures, social desirability, and successful completion were coded as binary variables, and all other dependent and independent variables were z-scored to report standardized Betas. ^1^Effect sizes for successful completion are reported as odds ratios. ^2^As only one participant both responded in socially desirable ways and completed training, this estimate is not meaningful. ^3^Performance covariates were only included in the model predicting obstacle course times, given that our question of interest regards which mindsets predict success of candidates entering training, rather than which mindsets predict success of those who made it through enough training to have performance outcomes. Models that include these performance covariates show consistent results and do not change the conclusions we draw from these findings (see Supplementary Material for further details). ^∗^*p* < 0.05; ^∗∗^*p* < 0.01; ^∗∗∗^*p* < 0.001.*

#### Do Failure-Is-Enhancing Mindsets Predict Success?

Candidates who reported a mindset that failure-is-enhancing had marginally less persistence in training (10% shorter; β = −0.14, *t*(148) = −1.86, *p* = 0.064, 95% CI [−0.30, 0.01]) and had a lower chance of completing phase 1 successfully (52% fewer candidates; *OR*(156) = 0.44, *p* = 0.012, 95% CI [0.23, 0.84]). We also find that candidates with a failure-is-enhancing mindset were slower on the Obstacle Course by 28 seconds (4.2% slower; β = 0.31, *t*(99) = −2.94, *p* = 0.004). The multiverse analyses suggest these results are analytically robust, finding consistent results on persistence (β_median_ = −0.22, *p*_median_ = 0.009), completion (*OR*_median_ = 0.57, *p*_median_ = 0.090), and obstacle course performance (β_median_ = 0.25, *p*_median_ = 0.016). We do not find evidence that failure mindsets predicted ratings by instructors or peers.

#### Do Non-limited Willpower Mindsets Predict Success?

We find that willpower mindsets predicted a greater number of negative comments by instructors per week (21% more, β = 0.28, *t*(130) = 2.86, *p* = 0.005, 95% CI [0.09, 0.48]), and a greater number negative evaluations by peers (76% more, β = 0.48, *t*(62) = 3.52, *p* < 0.001, 95% CI [0.21, 0.76]), but do not predict any other performance or success outcomes collected. The multiverse analyses suggest these results are analytically robust, finding willpower mindsets predicted a greater number of negative evaluations from instructors (β_median_ = 0.22, *p*_median_ = 0.026) and peers (β_median_ = 0.35, *p*_median_ = 0.005).

## Discussion

This research tested whether stress mindsets uniquely predict objective and subjective measures of success in the physically and mentally demanding environment of Navy SEAL training. We find candidates with greater stress-is-enhancing mindsets show improved performance on obstacle course times, last longer in the program, and are rated more positively by peers and instructors. These candidates do not show significantly greater completion rates, but this analysis may be underpowered given the binary nature of this outcome and low proportion of candidates completing the training. These findings were found to be robust across analytic models using multiverse analyses, and not dependent on particular exclusion criteria, covariates, or other analytic decisions.

To our knowledge, this is the first research to show stress-is-enhancing mindsets predict objective performance and success over a moderate length of time, in addition to less negative subjective evaluations by others. These findings suggest that stress-is-enhancing mindsets are relevant and impactful in extreme evaluative settings. We find that stress mindset predicts outcomes over and above a number of other baseline characteristics, including demographics (highest education level and mother’s education), fitness (Body Mass Index) and self-report individual differences (social desirability and optimism for success). In addition, our multiverse analyses found the effects to be robust to including particular covariates in the model. However, additional covariates potentially predictive of success (e.g., personality traits, prior training, and other relevant mental competencies; see Taylor et al., 2006) may give us greater insight into the unique predictive utility of mindset and warrant future research. Given the relatively small sample size, more research needs to be done to understand the robustness of these effects. We encourage researchers to include the stress-is-enhancing mindset measure as a predictor in studies of performance in stressful settings and situations to garner more evidence for its impact. One further limitation of the current research is that we were unable to collect proposed mechanisms by which stress mindsets influence outcomes, such as more frequent seeking of feedback for improvement, greater positive affect during stress, or adaptive hormonal responses to stress (Crum et al., 2013, 2017). Future studies should examine these psychological and physiological mechanisms, particularly during moments of stress, to better understand how stress mindsets increase performance.

We also find that failure-is-enhancing mindsets predicted worse outcomes in this setting. Candidates with a failure-is-enhancing mindset have slower obstacle course times, drop sooner from training, and have higher rates of dropout than their peers. These findings are inconsistent with literature suggesting positive beliefs about failure are beneficial in learning settings (Dweck and Leggett, 1988), but in line with past work done in evaluative settings (e.g., El-Alayli and Baumgardner, 2003). Failure is not highly tolerated in this particular setting, and those who fail early may be held to a higher standard to make up for prior failures. As one candidate noted upon making a mistake, “It was like sharks smelling blood in the water- the instructors saw that I was struggling and they swarmed.” Thus, it is perhaps unsurprising a failure-is-enhancing mindset may be detrimental when demands are increased due to failure and there is little opportunity to reflect on and learn from those failures. This is not to say failure-is-enhancing mindsets are detrimental in other portions of Navy SEALs training or in other aspects of candidates’ lives. When the focus is less evaluative later in training, we may discover the opposite pattern where these mindsets are beneficial, as found in longer-term educational settings (Blackwell et al., 2007).

We find no evidence that willpower mindsets predicted performance, persistence, or success in training. Yet it appears that holding a non-limited mindset may be linked to negative social consequences, as these candidates received more negative instructor and peer evaluations. One potential explanation is that a person with a non-limited willpower mindset may perceive others as having more available energy (see Francis et al., 2019) and thus may be less supportive of teammates who are struggling. A similar process has been found in the stress mindset literature, whereby those with greater stress-is-enhancing mindsets saw a struggling peer as undergoing less negative stress and thus less likely to offer help to this person (Ben-Avi et al., 2018). This relationship between non-limited willpower mindsets and negative evaluations from others might be masking an otherwise positive relationship between non-limited mindsets and persistence in training, particularly as instructors can make the setting more challenging for specific candidates by giving additional demands. Little research has focused on the social consequences of holding particular mindsets, which may be a fruitful avenue for future research.

This set of findings highlight the unique importance of stress mindsets in predicting objective measures of success in an intensely stressful setting. Although our focus on this unique population of Navy SEALs may limit the generalizability of our findings to typical stressors that most employees contend with, everyone encounters acute life stressors (such as having a child, a death of a loved one, or difficulties with a boss) that may impact their work performance and well-being (Vicino and Bass, 1978; Kobasa, 1979; see also Holmes and Rahe, 1967). Thus, we would suspect a similar (though perhaps less robust) effect of stress-is-enhancing mindsets on everyday work life.

Furthermore, this research may prompt organizations to create structures and opportunities that encourage stress-is-enhancing mindsets. We believe this could be incredibly impactful if done correctly, but more research is needed. Future collaborations between researchers and organizations should shed light on the causal link between mindsets and organizational success, as many stress-relevant programs in military, business, and educational settings have not undergone rigorous testing (see Richardson and Rothstein, 2008; Taylor et al., 2011). It is of the utmost importance to determine whether these mindsets can be taught to develop greater resilience, and future efforts should prioritize randomized-controlled trials to test these programs.

## Conclusion

Social and industrial-organizational psychology have made great strides in the last few decades to understand how to promote the success of employees and colleagues, and how mindsets can be powerful drivers of performance. However, many organizations have been promoting stress management or stress reduction techniques, rather than considering how mindsets about stress may impact performance. The current research suggests that stress-is-enhancing mindsets may be a powerful target for intervention, particularly in extremely demanding and stressful settings. We also find that certain mindsets predict unexpected negative outcomes in this particular context and propose that a mindset’s benefit is in part based on its interaction with the people, culture, and goals of the specific environment. This work contributes to the theoretical understanding of the boundary conditions for three impactful mindsets and provides further evidence that stress mindsets can influence outcomes across a range of performance, persistence and social measures. This study also highlights the need to better understand which mindsets may be most beneficial in a particular setting, and how organizations, managers, or employees can support adaptive mindsets that will help them succeed in the workplace and in their personal lives. Though future research is needed to fully test the causal role of mindsets, this study provides initial evidence that mindsets are too important to be left unacknowledged by organizational leaders. By identifying the impactful mindsets at play and recognizing the environmental influence on these mindsets, researchers and practitioners can make great strides in promoting people’s ability to thrive across the many domains and settings they encounter.

## Data Availability Statement

Due to privacy agreements with the Navy, we are unable to share the data used for this manuscript without prior approval from the Naval Health Research Center. If readers are interested in this dataset, we will support them in getting approval to access the data and would happily share the data once approval is secured. In lieu of these data sharing restrictions, the analysis scripts for all analyses reported and complete materials are publicly available to readers at the Open Science Framework (https://osf.io/sejym).

## Ethics Statement

The studies involving human participants were reviewed and approved by the Non-medical Human Subjects Research Institutional Review Board, Stanford University. Participants provided their written informed consent to participate in this study.

## Author Contributions

ES and AC developed the study concept. All authors contributed to the study design, supported the data collection, and approved the final version of the manuscript for submission. ES performed the data analysis and interpretation in collaboration with AC. ES drafted the manuscript. AC provided the critical revisions.

## Conflict of Interest

The authors declare that the research was conducted in the absence of any commercial or financial relationships that could be construed as a potential conflict of interest.

## References

[B1] Akinola M. Fridman I. Mor S. Morris M. W. Crum A. J. (2016). Adaptive appraisals of anxiety moderate the association between cortisol reactivity and performance in salary negotiations. *PLoS One* 11:e0167977. 10.1371/journal.pone.0167977 27992484 PMC5161466

[B2] Ben-Avi N. Toker S. Heller D. (2018). “If stress is good for me, it’s aprobably good for you too”: stress mindset and judgment of others’ strain. *J. Exp. Soc. Psychol.* 74 98–110. 10.1016/j.jesp.2017.09.002

[B3] Blackwell L. S. Trzesniewski K. H. Dweck C. S. (2007). Implicit theories of intelligence predict achievement across an adolescent transition: a longitudinal study and an intervention. *Child Dev.* 78 246–263. 10.1111/j.1467-8624.2007.00995.x 17328703

[B4] Blascovich J. Mendes W. B. (2010). “Social psychophysiology and embodiment”. in*Proceedings of the Handbook of Social Psychology*, eds Fiske S. T. Gilbert D. T. Lindzey G. 5th Edn, Vol. 1 (New York, NY: Wiley), 194–227.

[B5] Brooks A. W. (2014). Get excited: reappraising pre-performance anxiety as excitement. *J. Exp. Psychol. Gen.* 143 1144–1158. 10.1037/a0035325 24364682

[B6] Brown K. W. Ryan R. M. (2003). The benefits of being present: mindfulness and its role in psychological well-being. *J. Pers. Soc. Psychol.* 84 822–848. 10.1037/0022-3514.84.4.822 12703651

[B7] Crowne D. P. Marlowe D. (1960). A new scale of social desirability independent of psychopathology. *J. Consult. Psychol.* 24 349–354. 10.1037/h0047358 13813058

[B8] Crum A. J. Akinola M. Martin A. Fath S. (2017). The role of stress mindset in shaping cognitive, emotional, and physiological responses to challenging and threatening stress. *Anxiety Stress Coping* 30 379–395. 10.1080/10615806.2016.1275585 28120622

[B9] Crum A. J. Salovey P. Achor S. (2013). Rethinking stress: the role of mindsets in determining the stress response. *J. Pers. Soc. Psychol.* 104 716–733. 10.1037/a0031201 23437923

[B10] Duncko R. Cornwell B. Cui L. Merikangas K. R. Grillon C. (2007). Acute exposure to stress improves performance in trace eyeblink conditioning and spatial learning tasks in healthy men. *Learn. Mem.* 14 329–335. 10.1101/lm.483807 17522023 PMC1876756

[B11] Dweck C. S. Leggett E. L. (1988). A social-cognitive approach to motivation and personality. *Psychol. Rev.* 95 256–273. 10.1037//0033-295x.95.2.256

[B12] El-Alayli A. (2006). Matching achievement contexts with implicit theories to maximize motivation after failure: a congruence model. *Pers. Soc. Psychol. Bull.* 32 1690–1702. 10.1177/0146167206291946 17122180

[B13] El-Alayli A. Baumgardner A. N. N. (2003). If at first you don’t succeed, what makes you try, try again? effects of implicit theories and ability feedback in a performance-oriented climate. *Self Identity* 2 119–135. 10.1080/15298860309031

[B14] Epel E. S. McEwen B. S. Ickovics J. R. (1998). Embodying psychological thriving: physical thriving in response to stress. *J. Soc. Issues* 54 301–322. 10.1111/0022-4537.671998067

[B15] Francis Z. Seiber V. Job V. (2019). You seem tired, but so am I: willpower theories and intention to provide support in romantic relationships. *J. Soc. Pers. Relatsh.* 1 1–20. 10.1177/0265407519877238

[B16] Haimovitz K. Dweck C. S. (2016). Parents’ views of failure predict children’s fixed and growth intelligence mind-sets. *Psychol. Sci.* 27 859–869. 10.1177/0956797616639727 27113733

[B17] Harackiewicz J. M. Barron K. E. Tauer J. M. Carter S. M. Elliot A. J. (2000). Short-term and long-term consequences of achievement goals: predicting interest and performance over time. *J. Educ. Psychol.* 92 316–330. 10.1037//0022-0663.92.2.316

[B18] Holmes T. H. Rahe R. H. (1967). The social readjustment rating scale. *J. Psychosom. Res.* 11 213–218. 10.1016/0022-3999(67)90010-4 6059863

[B19] Jamieson J. P. Mendes W. B. Blackstock E. Schmader T. (2010). Turning the knots in your stomach into bows: reappraising arousal improves performance on the GRE. *J. Exp. Soc. Psychol.* 46 208–212. 10.1016/j.jesp.2009.08.015 20161454 PMC2790291

[B20] Job V. Dweck C. S. Walton G. M. (2010). Ego depletion—is it all in your head? implicit theories about willpower affect self-regulation. *Psychol. Sci.* 21 1686–1693. 10.1177/0956797610384745 20876879

[B21] Job V. Walton G. M. Bernecker K. Dweck C. S. (2013). Beliefs about willpower determine the impact of glucose on self-control. *Proc. Natl. Acad. Sci.* 110 14837–14842. 10.1073/pnas.1313475110 23959900 PMC3773743

[B22] Job V. Walton G. M. Bernecker K. Dweck C. S. (2015). Implicit theories about willpower predict self-regulation and grades in everyday life. *J. Pers. Soc. Psychol.* 108 637–647. 10.1037/pspp0000014 25844577

[B23] Jones M. Meijen C. McCarthy P. J. Sheffield D. (2009). A theory of challenge and threat states in athletes. *Int. Rev. Sport Exerc. Psychol.* 2 161–180. 10.1080/17509840902829331 PMC7016194 32116930

[B24] Kobasa S. C. (1979). Stressful life events, personality, and health: an inquiry into hardiness. *J. Pers. Soc. Psychol.* 37 1–11. 10.1037//0022-3514.37.1.1 458548

[B25] LePine J. A. Podsakoff N. P. LePine M. A. (2005). A meta-analytic test of the challenge stressor–hindrance stressor framework: an explanation for inconsistent relationships among stressors and performance. *Acad. Manag. J.* 48 764–775. 10.5465/amj.2005.18803921

[B26] McEwen B. S. Sapolsky R. M. (1995). Stress and cognitive function. *Curr. Opin. Neurobiol.* 5 205–216. 7620309 10.1016/0959-4388(95)80028-x

[B27] McNeish D. M. (2015). Using lasso for predictor selection and to assuage overfitting: a method long overlooked in behavioral sciences. *Multivar. Behav. Res.* 50 471–484. 10.1080/00273171.2015.1036965 26610247

[B28] Miao C. Qian S. Ma D. (2017). The relationship between entrepreneurial self-efficacy and firm performance: a meta-analysis of main and moderator effects. *J. Small Business Manag.* 55 87–107. 10.1111/jsbm.12240

[B29] Molden D. C. Dweck C. S. (2006). Finding “meaning” in psychology: a lay theories approach to self-regulation, social perception, and social development. *Am. Psychol.* 61 192–203. 10.1037/0003-066x.61.3.192 16594836

[B30] Motowidlo S. J. Packard J. S. Manning M. R. (1986). Occupational stress: its causes and consequences for job performance. *J. Appl. Psychol.* 71 618–629. 10.1037/0021-9010.71.4.618 3804934

[B31] Mueller C. M. Dweck C. S. (1998). Praise for intelligence can undermine children’s motivation and performance. *J. Pers. Soc. Psychol.* 75 33–52. 10.1037//0022-3514.75.1.33 9686450

[B32] Park C. L. Helgeson V. S. (2006). Introduction to the special section: growth following highly stressful life events—current status and future directions. *J. Consult. Clin. Psychol.* 74 791–796. 10.1037/0022-006x.74.5.791 17032084

[B33] Park D. Yu A. Metz S. E. Tsukayama E. Crum A. J. Duckworth A. L. (2017). Beliefs about stress attenuate the relation among adverse life events, perceived distress, and self-control. *Child Dev.* 89 2059–2069. 10.1111/cdev.12946 28872676 PMC5837904

[B34] Paunesku D. Walton G. M. Romero C. Smith E. N. Yeager D. S. Dweck C. S. (2015). Mind-set interventions are a scalable treatment for academic underachievement. *Psychol. Sci.* 26 784–793. 10.1177/0956797615571017 25862544

[B35] Podsakoff N. P. LePine J. A. LePine M. A. (2007). Differential challenge stressor-hindrance stressor relationships with job attitudes, turnover intentions, turnover, and withdrawal behavior: a meta-analysis. *J. Appl. Psychol.* 92 438–454. 10.1037/0021-9010.92.2.438 17371090

[B36] Podsakoff P. M. MacKenzie S. B. Lee J. Y. Podsakoff N. P. (2003). Common method biases in behavioral research: a critical review of the literature and recommended remedies. *J. Appl. Psychol.* 88 879–903. 10.1037/0021-9010.88.5.879 14516251

[B37] Podsakoff P. M. Organ D. W. (1986). Self-reports in organizational research: problems and prospects. *J. Manag.* 12 531–544. 10.1177/014920638601200408 8452065

[B38] Richardson K. M. Rothstein H. R. (2008). Effects of occupational stress management intervention programs: a meta-analysis. *J. Occup. Health Psychol.* 13 69–93. 10.1037/1076-8998.13.1.69 18211170

[B39] Romero C. Master A. Paunesku D. Dweck C. S. Gross J. J. (2014). Academic and emotional functioning in middle school: the role of implicit theories. *Emotion* 14 227–234. 10.1037/a0035490 24512251

[B40] Schneiderman N. Ironson G. Siegel S. D. (2005). Stress and health: psychological, behavioral, and biological determinants. *Annu. Rev. Clin. Psychol.* 1 607–628. 10.1146/annurev.clinpsy.1.102803.144141 17716101 PMC2568977

[B41] Stajkovic A. D. Luthans F. (1998). Self-efficacy and work-related performance: a meta-analysis. *Psychol. Bull.* 124 240–261. 10.1037/0033-2909.124.2.240

[B42] Steegen S. Tuerlinckx F. Gelman A. Vanpaemel W. (2016). Increasing transparency through a multiverse analysis. *Perspect. Psychol. Sci.* 11 702–712. 10.1177/1745691616658637 27694465

[B43] Taylor A. H. Schatz S. Marino-Carper T. L. Carrizales M. L. Vogel-Walcutt J. (2011). “A review of military predeployment stress tolerance training,” in *Proceedings of the Human Factors and Ergonomics Society Annual Meeting*, Vol. 55(Los Angeles, CA: SAGE Publications), 2153–2157. 10.1177/1071181311551449

[B44] Taylor M. K. Miller A. Mills L. Potterat E. Padilla G. A. Hoffman R. (2006). *). Predictors of Success in Basic Underwater Demolition/SEAL (BUD/S) Training-Part 1: What Do We Know and Where Do We Go From Here? (No. NHRC-06-27).* (San Diego, CA: Naval Health Research Center).

[B45] Tedeschi R. G. Calhoun L. G. (2004). Posttraumatic growth: conceptual foundations and empirical evidence. *Psychol. Inq.* 15 1–18. 10.1207/s15327965pli1501_01

[B46] Tibshirani R. (1996). Regression shrinkage and selection via the lasso. *J. R. Stat. Soc. Series B Methodol.* 55 267–288. 10.1111/j.2517-6161.1996.tb02080.x

[B47] Vicino F. L. Bass B. M. (1978). Lifespace variables and managerial success. *J. Appl. Psychol.* 63 81–88. 10.1037//0021-9010.63.1.81

[B48] Vohs K. D. Baumeister R. F. Schmeichel B. J. (2012). Motivation, personal beliefs, and limited resources all contribute to self-control. *J. Exp. Soc. Psychol.* 48 943–947. 10.1016/j.jesp.2012.03.002

[B49] Yeager D. S. Johnson R. Spitzer B. J. Trzesniewski K. H. Powers J. Dweck C. S. (2014). The far-reaching effects of believing people can change: implicit theories of personality shape stress, health, and achievement during adolescence. *J. Pers. Soc. Psychol.* 106 867–884. 10.1037/a0036335 24841093

[B50] Zion S. R. Schapira L. Crum A. J. (2019). Targeting mindsets, not just tumors. *Trends Cancer*. 5 573–576. 10.1016/j.trecan.2019.08.001 31706503 PMC7059887

