# Supplementary material for: Stress, Mindsets, and Success in Navy SEALs Special Warfare Training
Source: Front Psychol. 2020 Jan 15;10:2962. doi: 10.3389/fpsyg.2019.02962 (PMC6974804; doi:10.3389/fpsyg.2019.02962)
Supplement: Supplementary file 1 [file Data_Sheet_1.PDF]

Stress, Mindsets, and Success in Navy SEALs Special Warfare Training

Supplementary Material

1 Table S1: Zero-Order Correlation Matrix

	SM	FM	WM	Race	Edu	M_Edu	SD	BMI	SL	Completion	Persisted	OCourse	Peer-Neg	Peer-Pos	Inst-Demerit
Stress Mindset	1														
Failure Mindset	0.33	1													
Willpower Mindset	0.44	0.21	1												
Race (Non-White)	0.08	0.04	0.05	1											
Education (College Degree)	-0.02	-0.04	0.01	-0.03	1										
Mother Edu (College Degree)	-0.04	-0.07	0.02	0.03	0.22	1									
Social Desireability	0.25	0.03	0.3	0.1	-0.02	-0.05	1								
Body Mass Index	-0.01	0.02	0.11	0.08	0.27	0.05	-0.03	1							
Success LIkelihood	0.27	0.09	0.19	0.02	-0.07	-0.07	0.09	0.08	1						
BUD/S Completition	0.04	-0.13	-0.01	0.07	0.31	0.17	-0.11	0.15	0.13	1					
Weeks Persisted	0.1	-0.09	0.08	0.09	0.26	0.21	-0.13	0.2	0.18	0.71	1				
OCourse Time	-0.13	0.14	0.08	-0.07	-0.05	-0.1	0.04	-0.01	-0.15	-0.41	-0.55	1			
Peer-Negative Evals	-0.23	-0.13	0.26	-0.02	-0.16	-0.15	0.19	-0.24	-0.05	-0.17	-0.22	0.25	1		
Peer-Positive Evals	0.09	-0.15	-0.02	0.08	0.24	-0.05	-0.14	0.22	0.15	0.45	0.53	-0.39	-0.18	1	
Instructor Demerits	-0.19	0.05	0.12	-0.02	-0.18	-0.14	0.14	-0.03	-0.01	-0.19	-0.1	0.18	0.67	-0.34	1

Table S1: Pearson correlation coefficients between mindsets, baseline covariates, and outcome measures. Variable descriptions can be found in the main manuscript. Correlations with p-values > .10 are not filled with color. Correlations of with p-values <.10 are shaded from dark red for strongly negative correlations to dark blue for strongly positive correlations.

2 Observed scores of continuous measures

Measure	Possible Range	Observed Range	Mean	Median
Stress-is-Enhancing Mindset	1-6	2.62-6.00	4.54	4.5
Failure-is-Enhancing Mindset	1-6	1.00-6.00	4.81	4.83
Non-limited Willpower Mindset	1-6	2.33-6.00	4.5	4.5
Body Mass Index	NA	20-29	25.0	25
Perceived Likelihood of Success	1-5	1-5	4.55	5
Weeks Persisted	0-5	0-5	2.18	2
Obstacle Course Time (min)	NA	7.20-15.62	10.77	10.75
Peer- Negative Evaluations	0-72	0-22	2.46	1
Peer- Positive Evaluations	0-72	0-34	7.54	5.5
Instructor Demerits (per week)	NA	0-18	3.28	2.63

Table S2: Descriptive statistics for continuous measures collected. Variable descriptions can be found in the main manuscript.

3 Multiverse Analysis

As noted in the manuscript, the primary models reported above were based on theory and best methodological practices. To ensure our results were not due to a chance analytic decision, we perform 'Multiverse Analyses' (see Steegen, Tuerlinckz, Gelman, & Vanpaemel, 2016) in addition to the primary models. Below we present the results of 104,976 potential analyses we could have run in the "multiverse" of analytic models, which other reasonable researchers may have conducted when testing for effects on the same outcomes.

To run these analysis, we first identified some of the researcher degrees of freedom available to us (See Table S2 for summary). These broadly fall into four categories, 1) inclusion of demographic covariates, 2) inclusion of baseline survey covariates, 3) inclusion of early physical performance metrics, and 4) exclusion criteria for participants.

For each of the four demographic covariates, there were three main options for dealing with each of these items if we had randomly chosen an analytic model. We could have a) included demographics for all participants by imputing any missing demographic values to the mean value, b) included demographics in the model without imputing missing values, thereby removing participants with missing values, or c) not included the demographic as a covariate in the model. Race was not included in our reported models due to the lack of predictive validity in the cross-validated LASSO regression analyses. The three others were included in our primary models using imputed values to

maintain a larger sample for greater analytical power and ensure we were not systematically excluding participants who chose not to report demographic measures.

We had similar options for our potential survey covariates of setting-specific optimism, commitment to BUD/S, and having a mentor in the Navy. Based on our cross-validated LASSO regression analyses, we only included optimism for success in the analytic model. We excluded non-responses in our primary models, as these items were embedded in other survey measures and we suspected missing data in these items would reflect a participant not following instructions, rather than actively choosing to not report demographic information as above. For our social desirability measure, we used a cutoff criteria in our primary models (0 = fewer than six socially-desirable responses; 1 = six or seven socially-desirable responses), as we hypothesized answering zero to five of these question may reflect actual candidate characteristics rather than socially-desired reporting. However, we could have used a continuous measure of social desirability using the mean item score, or entirely excluded it from our model, and thus include these decisions in our multiverse models.

For early performance data, the main options were to exclude, include without missing values, and impute missing values to the mean. In our primary models, we only include these measures as covariates for obstacle course completion time during the first week of training, and excluded it in models predicting other dependent variables. The theoretical rationale for this is that we hypothesized that mindsets would be more predictive on the obstacle course if controlling for the more practiced measures of physical fitness as assessed by 2-mile swim and 4-mile run times. In addition, by excluding these covariates from other models, we could maintain a larger sample for greater analytical power, as performance measures were only collected for the subset of participants who made it through the first week of training.

In addition, there were three main potential exclusion criteria: 1) whether to remove outliers based on extreme responses to our three main mindset scales (>3 Standard Deviations away from the mean; $N = 3$), 2) whether to include those who dropped out of training due to medical reasons ($N = 15$), and 3) whether to include those who rolled to the next class; ($N = 12$). In the primary models, we include outliers of extreme responses on our mindset scales, as variation in these measures may still be meaningful to the underlying construct. However, it is a fairly common practice to exclude outliers in psychological research and thus include this choice in the multiverse analyses. We excluded participants who left due to medical reasons in the completion and persistence models, as medical issues do not necessary reflect the physical or mental toughness of candidates (e.g. medical drops may be due to accident or misfortune). However, a researcher might argue that a particular medical drop may be due to accident or misfortune, but that in aggregate these drops would still reflect the ability to achieve success in this setting, and thus we includes this choice in the multiverse analyses. We included candidates who “rolled” to the next class in our primary models, as we were most interested in successful completion with their class rather removal from the program entirely. A researcher might argue that these participants could be excluded because they neither fully failed out of training nor fully succeeded, so we included this choice in the multiverse analyses as well.

You can find a summary of multiverse analyses run for each mindset predictor and outcome in the graphs below. See figure caption for details.

3.1 Figure S1: Multiverse Analysis Effect Sizes for Stress Mindset Measure

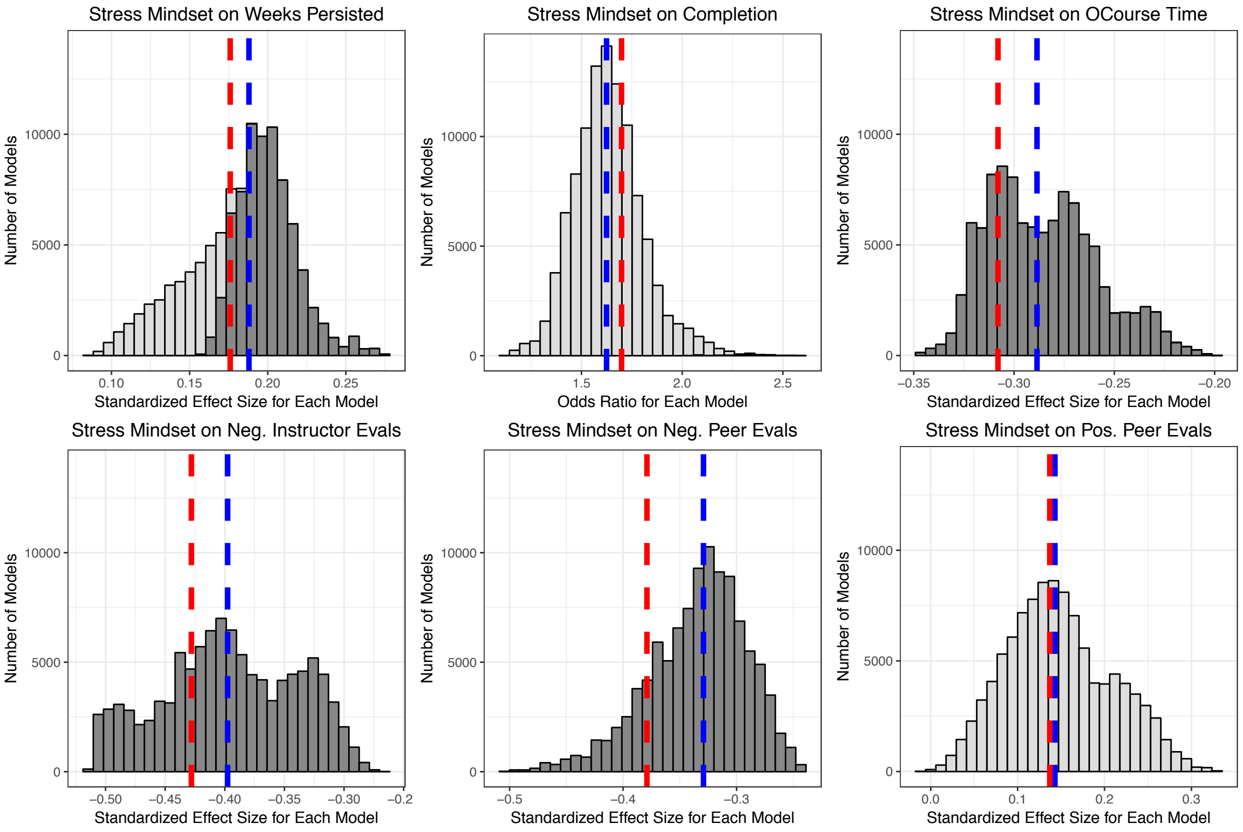

Figure S1. Each of the 104,976 individual model effect sizes is represented for stress mindset in the histograms above. The effect sizes of the primary models presented in the main manuscript are represented by the vertical red lines, and the medians of all the effect sizes found from the multiverse analysis are represented by the vertical blue lines. Models that were significant to the p<.05 level are shaded in a darker gray within each graph.

3.2 Figure S2: Multiverse Analysis Effect Sizes for Failure Mindset Measure

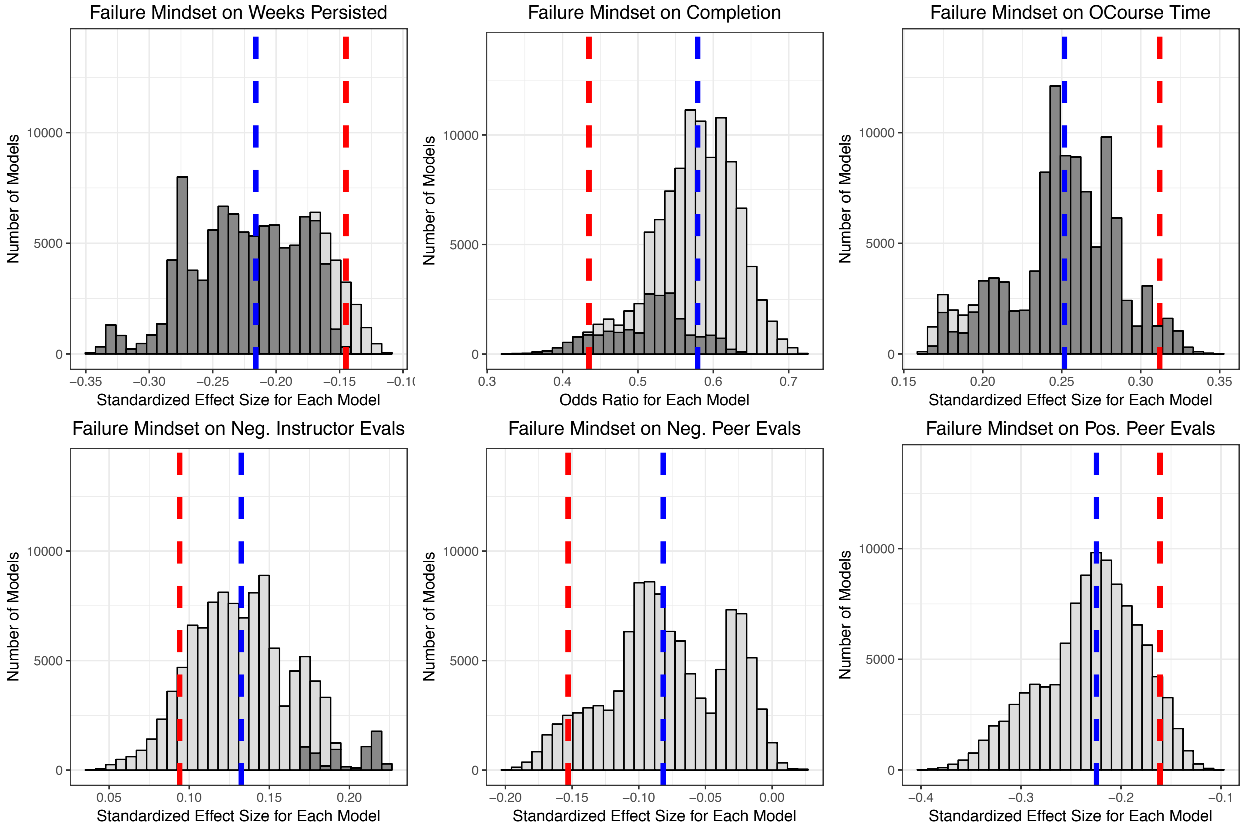

Figure S2. Each of the 104,976 individual model effect sizes is represented for failure mindset in the histograms above. The effect sizes of the primary models presented in the main manuscript are represented by the vertical red lines, and the medians of all the effect sizes found from the multiverse analysis are represented by the vertical blue lines. Models that were significant to the p<.05 level are shaded in a darker gray within each graph.

3.3 Figure S3: Multiverse Analysis Effect Sizes for Willpower Mindset Measure.

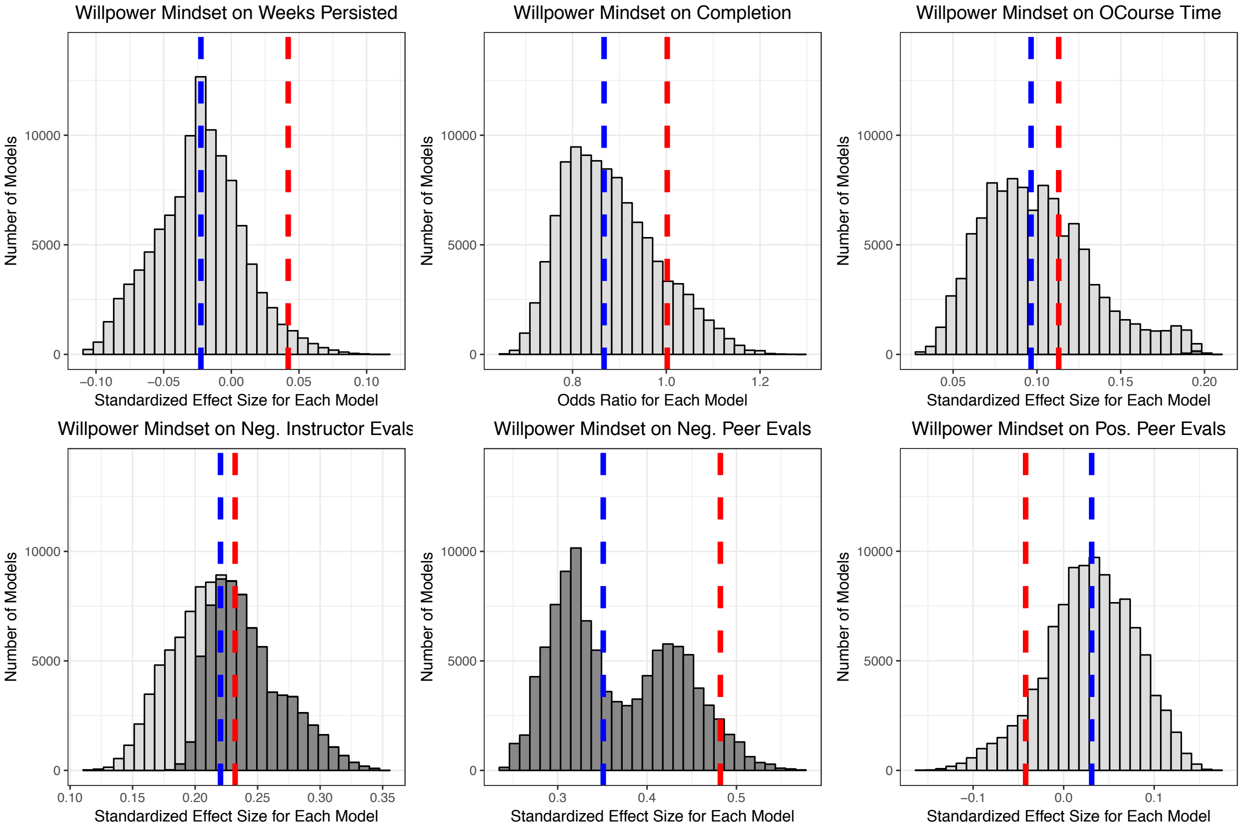

Figure S3. Each of the 104,976 individual model effect sizes is represented for willpower mindset in the histograms above. The effect sizes of the primary models presented in the main manuscript are represented by the vertical red lines, and the medians of all the effect sizes found from the multiverse analysis are represented by the vertical blue lines. Models that were significant to the p<.05 level are shaded in a darker gray within each graph.

4 Supplemental models including performance covariates

We chose not to include performance outcomes collected during the first week of training as covariates in our primary models predicting BUD/S success, persistence, and evaluations from others. This is because these performance metrics were only available for 111 of the 174 initial candidates who had not yet dropped from training. By including these indices as predictors in the model and dropping participants missing these performance times, we reduce the representativeness of our remaining sample, given that our hypotheses pertained to which mindsets predict success of candidates entering training, rather than which predict success for those who completed the first week of training and thus had performance measures available. Furthermore, attempting to impute this missing data may introduce additional statistical issues as this predictor is "missing not at random" and depends on the missing value itself. That is, those who would have had worse physical performance measures are more likely to be those whose data is missing, and it may be invalid to attempt to impute these values as predictors of success.

However, we believe models including performance covariates may be of particular interest for readers given that physical fitness is a pertinent predictor of success in this setting and we did not have baseline measures that adequately capture performance. As our goal of this research was to understand whether mindsets predict success above and beyond other predictors, we present these models with performance covariates (see Table S2 below). We find generally consistent findings with those reported in the main manuscript and we draw the same conclusions from these models as those reported in the manuscript.

	Primary Outcomes			Secondary Outcomes		
Measure	Successful Completion (logistic)[1]	Weeks Persisted	Obstacle Course Time	Instructor Evaluation (negative)	Peer Evaluation (negative)	Peer Evaluation (positive)
Demographic Covariates						
Education	3.860	-0.106	0.235	-0.043	0.044	0.209
Mother Education	0.961	-0.022	-0.132	-0.125	-0.326	-0.308
Body Mass Index	0.915	0.072	-0.089	-0.141	-0.227	0.072
Survey Covariates						
Social Desirability	0.000 [2]	-0.305	-0.110	0.064	0.483	-0.167
Optimism for Success	1.661	0.064	-0.051	0.073	-0.031	0.152
Performance Covariates						
4-Mile Run	0.382*	-0.191*	0.452***	0.410***	0.357**	-0.368**
2-Mile Swim	0.510	-0.357***	0.102	0.088	-0.110**	-0.371**
Mindsets						
Stress-is-Enhancing	1.899	0.211*	-0.308**	-0.322**	-0.359*	0.045
Failure-is-Enhancing	0.422*	-0.312***	0.312**	0.093	-0.166	-0.138
Non-limited Willpower	0.813	0.010	0.113	0.201	0.409**	0.075
N_{obs}	104	104	110	108	66	66
R^2	-	.448	.304	.317	.389	.445

Table S2. Standardized effect sizes for outcomes, including performance covariates of swim time and run time in each model. Education measures, social desirability, and successful completion were coded as binary variables, and all other dependent and independent variables were z-scored to report standardized Betas. [1]Effect sizes for successful completion are reported as odds ratios. [2]As only one participant both responded in socially desirable ways and completed training, this estimate is not meaningful. *$p < 0.05$; **$p < 0.01$; ***$p<0.001$.

5 Additional Survey Measures

Due to the opportunity to work with this unique population, additional measures not of primary interest to the current study were assessed to inform future work. These measures pertained to hypotheses not addressed in the current paper or exploratory in nature. All survey measures collected are described below and full survey materials can be found at (https://osf.io/sejym).

5.1 BUD/S Specific Mistake Mindsets

In addition to the broad mindsets assessed in the main manuscript, we collected an exploratory measure of mistakes-are-enhancing mindsets pertaining to the specific BUD/S training (four items; e.g. "Making mistakes during training is an important part of learning how to be a SEAL."). These items were created for the purpose of better understanding how global failure-is-enhancing mindsets relate to setting-specific beliefs. This scale was not validated and exploratory in nature, and unlikely to be informative to future research across other settings.

5.2 Perceptions of Training and SEALs Identity

We also included two sets of measures intended to determine whether the perception of BUD/S as an evaluative setting versus a training setting (five items; e.g. "BUD/S is designed to promote teamwork and group success.") impacts whether participants believed a SEALs identity is something innate or malleable over time (two items; e.g., "You are either SEAL material or you're not, and there is not much that can be done to change that."). In addition, participants answered open-response items that assessed similar questions (e.g. "What do you think the differences are between those that complete BUD/S and those that do not?").

5.3 Exploratory Willpower Measures

We were unsure of whether traditional nonlimited willpower measures would adequately capture participants' beliefs given the extreme nature of the training. Thus, we developed four additional willpower mindset items that explicitly asked about the ability to push beyond depletion e.g. ("Even if you think you've put all the energy you have into something, you can always find more."). These were secondary to our main mindset measures and we intended to analyze them only if we didn't see enough variance in the validated mindset scales. However, we find that these novel items had less variance than the previously establish scale, so we did not analyze them for the purposes of this paper.

5.4 Perceived Stress

The Perceived Stress Scale (PSS; Cohen, Kamarck, & Mermelstein, 1983) is a measure of global perceptions of stress in participants' lives, particularly how much they feel their lives are unpredictable, uncontrollable, or overwhelming. These items do not measure the amount of stressful events, but rather the positive and negative cognitions surrounding these stressful events ("In the last month, how often have you felt that you were on top of things?"; "In the last month, how often have you been angered because of things that were outside of your control?" [reverse-coded]). Participants reported how much they agree with 10 statements (1 = "Strongly Agree"; 6 = "Strongly Disagree"). We had initially planned to analyze these items as a covariate or moderator of stress mindset on outcomes, as past work has suggested those with greater distress may benefit more from stress-is-enhancing mindsets. The reason we have chosen not to include PSS in the main text is primarily because the PSS was measured at baseline and asked participants to reflect on the past month (before

training began). While stress prior to BUDS may impact performance in an accumulative fashion, we felt it was not a good marker of the amount of stress felt during the training. In addition, because of this unique setting, candidates were exposed to similar amount of stress in a tightly controlled environment, further suggesting that stress mindset predicted success during similar levels of stress.

Regardless of this limitation, we recognize that the impact of candidates' perceived stress prior to training may still be of interest to many readers. Thus, we ran additional analyses on the PSS as a predictor of success, and as a moderator of stress mindset on success. We find that when we add PSS as a predictor of persistence (weeks until dropping out of training), it does not add significant predictive validity to the model ($p = .61$). However, with its inclusion, we find stress mindsets appear to be directionally more significant as a predictor of persistence compared to the model reported in the paper ($\beta = 0.21$, $t(140) = 2.41$, $p = .017$, 95% CI [.04, .39]), but this may in part be due to excluding 7 participants who did not complete PSS measures at baseline and were excluded from the analysis sample.

In addition, we find a marginal interaction between baseline PSS and stress mindset on persistence ($\beta = 0.14$, $t(139) = 1.77$, $p = .080$, 95% CI [-.02, .30]), such that for those with high levels of PSS (+1 SD), stress-is-enhancing mindset was greatly predictive of persistence through BUD/S ($\beta = 0.38$, $t(139) = 2.95$, $p = .004$, 95% CI [.12, .63]), whereas those with low levels of PSS (-1 SD), stress-is-enhancing mindset was not significantly predictive of persistence through BUD/S ($\beta = 0.09$, $t(139) = 0.80$, $p = .42$, 95% CI [-.13, .31]).

Although this marginal interaction should be interpreted with caution, these results do converge with current theories on stress-is-enhancing mindsets, in that we'd expect these mindsets to be most predictive for those undergoing the greatest amount of stress (Crum, 2013; Crum, Handley-Miner, & Smith, in press).

5.5 Follow-up Surveys

In addition to the baseline surveys given to participants, two additional surveys of measures were collected. The first was given directly before week 4 (referred to as "Hell Week"). This was intended to test whether beliefs at baseline (as tested in the current manuscript) or perceptions of the training process would be better predictors of eventual success in BUD/S Phase One. However, as only 44 of the participants made it to week 4, these analyses were underpowered and were not formally analyzed. The second survey was intended to be given to candidates after being removed from the class, either from dropping, rolling to the next class, or upon graduation. This was intended to test two primary research questions not related to the current article: 1) Do candidates' beliefs change throughout training? and 2) Does the direction of this change predict when candidates will drop out of training?
